# Research on the mechanism of perceived value of cultural and creative products on consumers’ purchase intention

**DOI:** 10.1371/journal.pone.0343563

**Published:** 2026-03-06

**Authors:** Xiaofei Lu, Shouwang Li, Zeqian Wang

**Affiliations:** 1 School of Art, Xi ‘an University of Science and Technology, Xi ‘an, Shaanxi, China; 2 School of Management, Xi ‘an University of Science and Technology, Xi’ an, Shaanxi, China; USTC: University of Science and Technology of China, CHINA

## Abstract

In order to resolve the paradox of “high cultural value, low market conversion” and the homogenisation dilemma of cultural and creative products markets, this study proposes an extended analytical framework for cultural and creative products, based on the theory of *Customer Perceived Value.* The study systematically examines the impact of consumer perceived value on purchase intention from six dimensions: functional quality value, function price value, emotional value, social value, perceptual innovation, perceptual cultural, customer satisfaction, and purchase intention. A questionnaire survey of consumers at the Shaanxi Provincial Museum was conducted, yielding 333 valid responses. These responses were analysed using structural equation modelling. The study revealed that functional quality value, function price value, emotional value, and perceptual innovation all significantly positively influenced customer satisfaction, indirectly driving purchase intent through the full mediation effect of satisfaction. However, the impact of social value did not reach statistical significance. This research challenges the conventional limitations of traditional perceived value models, unveiling a chain mechanism in cultural and creative consumption: The sequence of events leading to the formation of purchase intent is as follows: first, the customer must perceive the value of the product; second, they must be satisfied with this perception. The findings provide a theoretical basis for understanding the pivotal role of cultural value in the transformation of cultural phenomena into market behaviour. The practical implications of this theory suggest that the development of cultural and creative products should move beyond the simple accumulation of symbols. By innovatively designing cultural elements to enhance their perceptibilities and leveraging narrative experiences to improve cultural interpretability, products can strengthen practical functionality and emotional resonance while simultaneously boosting consumers cultural identity and appreciation of innovation. This approach has been demonstrated to effectively activate purchase intent.

## 1. Introduction

In the context of China's “14th Five-Year Plan for Cultural Industry Development,” which explicitly advocates for the integration of digitalisation and industrialisation in the cultural industry, museums and cultural heritage sites have emerged as a pivotal policy and practical direction. These institutions have been instrumental in catalyzing cultural consumption through the implementation of creative design. In the context of the policy initiative, museums and heritage sites throughout the country have introduced a range of creative products, with the objective of stimulating consumer demand through the integration of innovative designs with a profound cultural foundation. As the cultural industry continues to flourish, these products have emerged as vital conduits of cultural heritage and innovation. Indeed, they have become a pivotal element within the consumer market due to their innovative formats, rich content, and extensive appeal [1]. However, beneath the surface of industrial prosperity, deep-seated structural challenges have become increasingly evident. Issues such as product homogenization, superficial symbolic replication, and a lack of substantive innovation are widespread, leading the market into a predicament of “high cultural value, low market conversion.” In order to identify the underlying causes, this study conducted a preliminary survey of consumers in cultural and creative spaces across multiple museums in Shaanxi Province. Furthermore, a significant proportion of respondents identified current products as suffering from “symbolic clutter” and “lack of practicality.” It is noteworthy that homogenization has accelerated consumer interest decline by approximately 42% compared to conventional goods. This predicament underscores the existing model's notable deficiencies in eliciting profound consumption aspirations and expediting the economic conversion of cultural value. These practical challenges reveal critical gaps in academic research regarding the mechanisms of cultural and creative consumption behaviour.

Whilst the Customer Perceived Value (CPV) theory provides a classic framework – “perceived value→satisfaction→purchase intention”–for understanding general product purchase intentions, its direct application to cultural and creative products faces theoretical limitations in explanatory power. The crux of the issue pertains to the inability of the conventional CPV framework, which places significant emphasis on the functional, emotional and social value dimensions, to adequately capture the dual nature of cultural and creative products as both “cultural carriers” and “creative commodities.” Existing research frequently exhibits three major limitations: Firstly, the absence of value dimensions is evident. Current frameworks place excessive emphasis on generic value indicators, such as the five-dimensional model of function/ emotion/ society/ cognition/ condition proposed by Sheth et al. [2]. However, this approach overlooks the distinctive “cultural decoding needs” of cultural and creative products. When consumers evaluate a notebook featuring bronze artifact patterns, their assessment involves not only paper quality (functional value) or esthetic experience (emotional value), but also the need to interpret the deeper meaning of cultural symbols. This “cultural perception” is in fundamental opposition to the symbolic value of traditional commodities. Second, the mediating mechanism remains unclear: While existing research suggests customer satisfaction (CS) mediates the relationship between perceived cultural value (CPV) and purchase intention (PI) [3], it fails to address the unique situational effects of cultural and creative products. Moreover, although most scholars incorporate social value into the CPV framework, empirical evidence regarding whether cultural product purchases are driven by “social image enhancement” or “cultural identity” remains scarce [4]. Survey data reveals that among customers of the Palace Museum's cultural and creative flagship store, 38% of repurchases stem from the psychological motivation of “completing cultural narratives,” suggesting that the academic community should re-examine the nonlinear pathways through which cultural identity influences satisfaction formation. Third, insufficient localization adaptation: Western-dominated value scales [5] struggle to capture Chinese consumers “gift-giving situational preferences.” Data from the Shaanxi History Museum shows that 65% of cultural and creative products are sold as souvenirs, where the core of social value is not personal image building but “cultural capital transmission,” which significantly deviates from the definition of social value proposed by Hollenstein and Colasante [6]. Existing models have thus far been unable to identify which specific value dimensions are not being effectively transmitted, leading to “obstructions” in the chain from cultural identity to purchase decisions. Furthermore, the CPV framework, which is derived from Western consumption contexts [5], has been shown to have limitations in its ability to adapt to local contexts when explaining the unique “cultural inheritance” motivations and “gift context” preferences of Chinese consumers. In this context, the exploration of the mechanisms underpinning cultural product consumption behaviour has emerged as a pivotal area of shared concern within both academic and industry circles.

In order to address the aforementioned theoretical challenges, this study systematically expands the Customer Perceived Value (CPV) theory to develop an integrated framework for analysing cultural and creative products. The framework combines two core dimensions – ‘cultural perception’ and ‘innovation perception’ – and empirically validates its pivotal role in the ‘perceived value → customer satisfaction → purchase intention’ pathway. This generates three theoretical contributions. Firstly, this study achieves a groundbreaking extension of theoretical frameworks in the value dimension. The present study proposes and validates that “cultural perception” and “innovation perception” constitute independent and pivotal value dimensions driving cultural and creative consumption, thus transcending the conventional paradigm of CPV models that primarily rely on functional, emotional, and social values. The former measures consumers depth of interpretation and identification with embedded cultural symbols in products, while the latter evaluates their perception of design's creative transformation. This research signifies a paradigm shift from a general model to a culturally specific framework for consumption analysis. Secondly, this study elucidates the complete mediating pathway of cultural value transformation in terms of its mechanism. Utilising structural equation modelling, we systematically examined the driving effect of the extended CPV framework with new dimensions on satisfaction, rigorously verifying the mediating role of satisfaction between perceived value and purchase intention. The particular significance was the quantification of differential impact weights across value dimensions, which revealed a hitherto unacknowledged key mechanism: that profound cultural value cannot directly translate into purchasing behaviour. Instead, it is necessary to stimulate consumers holistic satisfaction experience to achieve effective conversion. This provides a precise theoretical explanation for resolving the industry paradox of “high cultural value, low market conversion.” Thirdly, it provides empirical guidance for localized design strategies in practical contexts. The study draws upon the established cultural consumption paradigms of China, asserting that efficacious cultural and creative development must transcend mere superficial symbolic transplantation. The core strategy underpinning this approach involves the enhancement of cultural elements through innovative design, and the facilitation of the interpretation of cultural significance via situational narratives. This dual objective is intended to simultaneously drive consumers cultural identity and their innovative appreciation. This offers practitioners a practical framework for transitioning from the initial stages of value creation and transmission to the subsequent phase of value realization, thereby facilitating the transformation of cultural resources into market-compliant products.

## 2. Theoretical basis and literature review

**Fig 1:**
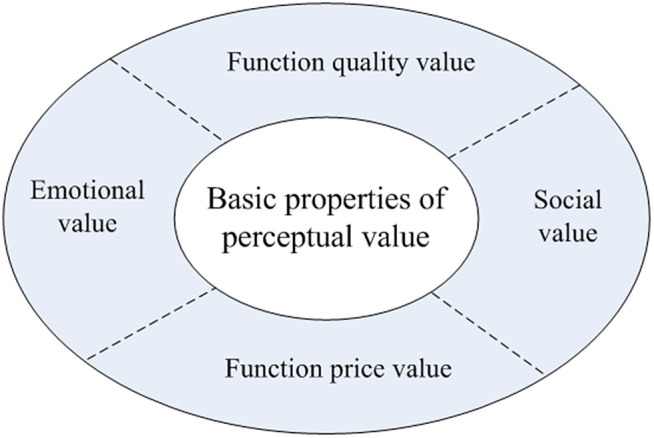
Basic attributes of perceived value.

The term “perceived value” is employed to denote the balance between benefits and costs that is perceived by consumers when purchasing a product [7]. A multi-dimensional analysis of psychological factors influencing consumer decisions has been developed by scholars, forming the core of the Perceived Value Theory. Despite the fact that this categorization approach has gained widespread academic recognition, variations emerge across research domains. For instance, Sheth et al. [2] investigated how social, emotional, functional, cognitive and situational factors influence the decision-making stages prior to and following purchases. The PERVAL scale, developed by Sweeney and Soutar [3], refined its dimensions into four operational categories: social value, emotional value, functional quality, and price value (see Fig 1). This framework subsequently became a widely adopted foundational tool in empirical research. These classic models form the theoretical basis for understanding the “perceived value-satisfaction-purchase intention” chain mechanism in general commodity consumption. These classical models constitute the theoretical foundation for understanding the chain mechanism of “perceived value-satisfaction-purchase intention” in general commodity consumption. In the context of the cultural and creative industries, scholars have endeavored to implement the CPV framework within this specialised domain. There is a broad consensus that the evaluation of the economic worth of cultural and creative products cannot be constrained by conventional functional categories. For instance, Gounaris et al. [8] proposed a six-dimensional model encompassing product, process, humanistic, emotional, social value, and perceived sacrifice, demonstrating its positive correlation with satisfaction. As Shu and Shao [9] emphasised, innovation, education and the experiential dimensions are of importance in the context of cultural consumption. Chen and Lei [10] proceeded to provide a more explicit introduction to the “cultural value” dimension. The extant literature suggests that high-quality cultural and creative products generally establish a symbiotic value relationship characterised by “functional foundation, cultural experience, and emotional connection” [11], driving the industry's transition from souvenir sales to cultural services.

However, the direct application of the CPV framework derived from traditional commodities to cultural and creative products reveals fundamental theoretical contradictions. The fundamental issue pertains to the irreconcilable conflict between static, universal value dimension classifications and the dynamic, context-dependent nature of cultural and creative consumption. Firstly, while extant research has expanded the dimensionality of the model. For example, Sheth et al. [2] incorporating contextual value and Sweeney and Soutar [3] optimizing operational feasibility – the dimensional system still fails to adequately account for the structural influence of cultural fields on value construction. The value of cultural and creative products is derived not only from their practical functions (functional value) or social recognition (social value), but more fundamentally from their significance as cultural symbols Peris-Ortiz et al. [12]. As Borisova [13] highlighted, the added value of these products is contingent not on the labour input involved in their production, but rather on consumers subjective evaluation of how well they meet their personal cultural needs. This evaluation is closely tied to consumers ability to interpret and resonate with the “cultural codes” embedded in the product, a dimension that traditional frameworks fail to capture. Secondly, cultural and creative products possess a dual value generation mechanism in that they serve as both carriers of cultural meaning and products of innovative design [14]. The symbolic and creative nature of these systems [15] constitutes their fundamental characteristics.Whilst extant CPV frameworks offer partial explanations of the practical (functional value) and self-pleasure (emotional value) dimensions, they systematically overlook two critical value sources: The terms “cultural interpretation” and “innovation appreciation” are defined as follows: For instance, although research on museum cultural and creative products has addressed factors like emotional resonance, aesthetic value, and cultural connotation [16],these aspects remain fragmented across different dimensions [17]. Theoretical integration and elevation of these elements into core CPV components have yet to be achieved.

The theoretical contradictions previously referenced manifest in practice as an industry paradox characterised by “high cultural value but low market conversion,” accompanied by significant challenges such as product homogenisation and symbolisation (as evidenced by museum cases in Shaanxi, Liaoning, and other regions). Despite the exploration of purchasing factors from a consumer perspective by scholars (e.g., authenticity and hedonism [18], or the integration of cultural elements through models [19], the majority of studies continue to treat culture or innovation as external contexts or marketing elements rather than intrinsic dimensions of value perception [20]. This approach, however, does not adequately address the fundamental question of whether the use of these materials is beneficial or detrimental to the learning process. Which unique value perception dimensions directly drive satisfaction and purchase intention in cultural and creative consumption?

Consequently, traditional CPV models face dual limitations in the cultural and creative sector: The terms “cultural absence” and “innovation absence” are employed. In order to address these challenges, it is essential to make targeted adjustments and undertake systematic restructuring of theoretical frameworks. Addressing this gap, the present study proposes integrating “cultural perception” (measuring depth of interpretation and identification with cultural symbols) and “innovation perception” (evaluating appreciation of creative design transformations) as core dimensions into the CPV framework. This integration is not merely an addition, but rather an attempt to construct a unified model that simultaneously encompasses both the “cultural carrier” and “creative product” attributes of cultural and creative goods. The objective is to more accurately reveal their value generation and transformation mechanisms, thereby establishing a new theoretical foundation for understanding consumer behaviour [21] and providing design optimisation recommendations [22].

## 3. Research hypotheses

### 3.1. Research hypotheses

Oliver [23] observed that the discrepancy between the actual consumption experiences and expectations, termed “customer satisfaction,” exerts a positive or negative influence on subsequent purchase intentions. Konuk [24] investigated consumer patterns and confirmed a significant positive correlation between perceived value and customer satisfaction. The analysis indicates a direct correlation between the perceived value of cultural and creative products and customer satisfaction levels. Furthermore, it is evident that enhanced satisfaction has a positive impact on the overall consumption experience.

#### 3.1.1. Basic dimensions of cultural and creative products.

The concept of customer satisfaction is determined by the congruence between the actual experiences of the customer and their expectations. The functional quality value is indicative of a product's practicality and reliability. The occurrence of positive satisfaction is contingent upon the fulfillment of customer expectations in the context of cultural and creative products. Such expectations may pertain to the durability of materials, the esthetic appeal of designs, and the precision with which cultural elements are represented. Conversely, the experience of dissatisfaction arises in instances where these expectations are not met. As posited by Saleem et al. [25], the hypothesis that “perceived product quality positively influences purchase intention” was proposed. Utilizing Likert 5-point scales to assess users perceptions prior to and following the acquisition process, the researchers identified a partial mediating effect between customer satisfaction and purchase intention with regard to perceived value. This finding aligns with the findings of D.G. Khan's research, which examined regional preferences for durable, reliable, and sustainable high-quality products. Chaerudin and Syafarudin [26] conducted a study to investigate the impact of product quality, service quality and price on medical device procurement decisions and user satisfaction (see reference for study details). The findings of the study demonstrated that all three variables significantly influenced consumer satisfaction, with price having the most significant impact.

A substantial body of research has demonstrated a positive correlation between product quality and consumer purchase intention. The quality of function, as the core attribute of products, exerts a direct influence on customers evaluations of cost-effectiveness. It is imperative that cultural and creative products strike a balance between cultural expression and practicality, as deficiencies in functional quality have the potential to diminish the appeal of cultural value-added products. Moreover, these products fulfill dual roles as both cultural carriers and practical tools. The failure of both “cultural symbols” and “practical tools” can be attributed to poor functional quality, which has a significant impact on satisfaction levels. Consequently, the functional quality value serves as a fundamental driver of satisfaction by fulfilling customers basic need for “practicality”. It is evident that consumers are able to recognize the product's fundamental utility to a greater extent when the functional quality is elevated, thus resulting in heightened satisfaction. It is from these findings that the following hypotheses are proposed:

*H1a: The function quality value of cultural and creative products significantly improves customer satisfaction.*

Albari and Kartikasari [27] advanced the hypothesis that price functions as an indicator of quality level. In order to align product features and services with consumer perceptions, pricing must be compromised – a finding that demonstrates the positive correlation between price and customer satisfaction. Razak et al. [28] further demonstrated the impact of pricing on customer satisfaction. In this study, the term “price” is defined as functional value.

The functional price value relationship is indicative of consumers perception of “cost-benefit alignment”. It is evident that elevated cost-effectiveness has a propensity to engender heightened levels of satisfaction. Conversely, the presence of either exorbitant premium pricing or the provision of low-quality products at reduced rates has been shown to engender dissatisfaction. Consequently, it is imperative that the pricing of cultural and creative products is aligned with their functional quality. Exorbitant prices may result in customers perceiving the “cultural premium as unreasonable”, whereas excessively low prices may engender concerns regarding product quality or cultural authenticity. Furthermore, it is imperative for cultural and creative products to strike a balance between cultural premium and functional value. For instance, if the cultural calendar of the Palace Museum is priced excessively without the implementation of practical innovations, customers may perceive that the “cultural symbol” does not merit the cost. Conversely, a combination of reasonable pricing and practical functionality has been shown to significantly boost satisfaction. It can thus be concluded that the relationship between functional price value and satisfaction is direct, with the former influencing consumers perception of “cost-effectiveness fairness”. It is evident that when customers perceive that “the functional value obtained through payment is reasonable”, there is a substantial increase in satisfaction. The following hypotheses are thus proposed by this paper:

*H1b: The function price value of cultural and creative products significantly improves customer satisfaction.*

The fundamental nature of cultural and creative products is predicated on the delivery of emotional experiences. The emotional value of these experiences has been shown to satisfy customers spiritual needs, thus transcending material functions to create a more profound sense of satisfaction. Specifically, these products evoke emotional resonance through cultural symbols (e.g., a sense of belonging or national pride). It has been demonstrated that a higher emotional value is associated with a stronger psychological attachment and greater customer satisfaction. Nurfaedah and Mustikasari [29] demonstrated the positive impact of emotional value on customer satisfaction, thereby providing a valuable contribution to the field. Furthermore, the enhancement of customer satisfaction through the process of “cultural identity labeling” has been demonstrated, thereby offering a potential solution to the challenges posed by functional and price-related shortcomings. Sheth et al. [2] and colleagues were the first to introduce the concept of emotional value within the theoretical framework of consumption value, analyzing factors such as anxiety, security, and confidence in cigarette purchasing decisions. A substantial body of research has demonstrated that emotional value is the principal factor that differentiates smokers from non-smokers. Consequently, emotional value fulfills higher-level psychological needs, fostering emotional attachment and unique experiences that substantially boost satisfaction. The paper puts forward the following hypotheses:

*H1c: The emotional value of cultural and creative products significantly improves customer satisfaction.*

The social value of cultural and creative products is manifested through two mechanisms: “group belonging” and “social status symbolism”. For instance, the purchase of high-end cultural gifts has been demonstrated to satisfy the identity recognition needs of “cultural elites” and enhance satisfaction. It is evident that certain products are able to leverage premium pricing and cultural scarcity, thus becoming vehicles for “conspicuous consumption”. In such cases, the social value of the product directly impacts customer satisfaction. It is evident that even in the context of price increases, collaborations with luxury brands continue to garner enthusiastic buyers, underscoring the viability of affordable items. Furthermore, the utilization of products that embody social values, such as eco-friendly materials or support for intangible cultural heritage artisans, fosters a sense of moral fulfillment in customers through their participation in social welfare initiatives, thereby enhancing satisfaction. Consequently, social value fulfills societal demands, such as “social image management” and “moral responsibility fulfillment”, thereby enhancing symbolic utility and significantly influencing satisfaction. In his research, Sweeney and Soutar [3] proposed the social value dimension, providing an explanation of its impact on consumer purchasing behavior. The following hypothesis is thus formulated:

*H1d: The social value of cultural and creative products is significantly improved and customer satisfaction is increased.*

#### 3.1.2. The unique attributes of cultural and creative products.

In accordance with the principles of innovation diffusion theory, innovative products have the capacity to attract consumer attention through their novelty, thereby stimulating their desire to explore. Consequently, the innovation of cultural and creative products is closely related to their originality. From the perspective of the consumer, that product innovation in the retail industry fulfills consumers psychological need for novelty and uniqueness, enhances emotional experiences (e.g., surprise and pleasure), and positively impacts satisfaction. Moreover, Naveed et al. [30] further corroborated the role of innovation in functional products, contending that innovative designs can optimize product utility and reduce consumers “esthetic fatigue” toward traditional products. It is evident that the innovation of cultural and creative products is not solely reflected in visual design, but is also achieved through functional innovation, thereby creating differentiated value. A notable example of this synthesis of practicality and artistry can be found in the “Thousand Mile Long River and Mountains” notebook by the Palace Museum. This “dual innovation” may result in an increased level of consumer recognition of the product, thereby enhancing satisfaction.

As posited by Wikhamn [31], innovation exerts an indirect influence on satisfaction, operating through the fulfillment of consumers personalized needs. The target demographic for cultural and creative products frequently seeks products that are unique, and the perception of innovation can enhance their sense of fulfillment in the domain of “self-expression”. For instance, the Dunhuang Academy's “Feitian” themed cosmetics blend traditional culture with modern esthetics, thereby preserving cultural essence while meeting contemporary trends. Such innovations have been found to resonate more emotionally with female consumers. Furthermore, innovation and cultural relevance are not mutually exclusive – moderate innovation is more market-acceptable than radical innovation. The National Museum's “Cultural Relics Cookies” serve as a prime example of this integration, where the incorporation of bronze artifact patterns into food design not only preserves cultural symbols but also introduces new functionalities. This “culture + innovation” approach may be instrumental in achieving optimal consumer satisfaction. The paper puts forward the following hypotheses:

*H2: The perceptual innovation of cultural and creative products significantly improves customer satisfaction.*

The inspiration for museum cultural and creative products primarily stems from traditional culture, where the selection and application of cultural elements directly impact user satisfaction [20]. Jin Hoare and Butcher [32] research on Chinese cultural values revealed that consumers’ identification with cultural symbols fosters a sense of “belonging,” thereby enhancing their satisfaction and loyalty. As a case in point, the cultural and creative products of the Palace Museum, with their emphasis on imperial elements, have been shown to evoke consumers’ emotional memories of historical culture, thereby reinforcing their cultural pride. Specifically, cultural elements can be analyzed into explicit components (e.g., patterns and materials) and implicit connotations (e.g., values and spirit). Explicit elements are known to convey cultural messages directly, while implicit connotations have been shown to influence satisfaction through emotional resonance. The Suzhou Museum's “Wen Zhengming’s Hand-Planted Wisteria” derivative product serves as a pertinent example. This product not only replicates the botanical form (explicit element) but also conveys the refined tastes of the literati (implicit element). This dual-layered cultural approach has the potential to enhance consumer satisfaction.

Moreover, the cultural sophistication of creative products frequently depends on the endorsement of authoritative cultural institutions, as consumers often perceive cultural value to be synonymous with product quality assurance. Within the context of Chinese culture, consumers exhibit stronger initial trust in products endorsed by cultural credentials. This trust has been demonstrated to reduce perceived purchase risks and, by extension, enhance satisfaction. For instance, Dunhuang Academy-authorized cultural products have been shown to engender greater consumer confidence on account of their “official certification” attribute. The following hypotheses are proposed by this paper, based on the analysis:

*H3: The perceptual culturality of cultural and creative products significantly improves customer satisfaction.*

### 3.2. Customer satisfaction and purchase intention

The likelihood of consumers choosing to purchase a product after gaining knowledge about it is termed “purchase intention.” The Expectation Confirmation Theory postulates that satisfaction is derived from the congruence between real experiences and anticipated outcomes [33]. Elevated satisfaction levels have been demonstrated to engender repurchase intentions. Recent studies have demonstrated that customer satisfaction is a fundamental predictor of purchasing intent. This notion is further substantiated by the findings of Dash et al. [34], which revealed a substantial positive correlation between customer satisfaction and purchase intention within market consumption patterns. This finding extends to cultural and creative products. As commodities that combine practicality and cultural attributes, customer satisfaction is derived from functional aspects such as quality and design, as well as cultural immersion and emotional resonance. When consumers find themselves satisfied with the cultural value, innovative design, and user experience of these products, they will develop positive cognitive evaluations that enhance repurchase or referral tendencies. Moreover, the consumption of cultural and creative products frequently entails emotional investment, encompassing aspects such as cultural identity and esthetic fulfillment. It is evident that elevated levels of satisfaction have the potential to reinforce brand trust and loyalty, thereby exerting a direct influence on purchase intentions.

Moreover, the prevailing cultural identity theory posits that the cultural attributes of cultural and creative products may serve to reinforce the correlation between satisfaction and purchase intention [35]. The direct impact of satisfaction on purchase intention can be illustrated as follows: The establishment of positive emotional connections is a consequence of high satisfaction, which in turn enhances consumers trust in the product. This, in turn, has a positive effect on their willingness to make a purchase. When the cultural essence of a product aligns with the needs of the user, the satisfaction-driven effect is particularly pronounced. The following hypotheses are proposed by this paper:

*H4: The customer satisfaction of cultural and creative products significantly improves the purchase intention.*

### 3.3. Mediating role of customer satisfaction

Saha et al. [36] demonstrated that online shopping intention is significantly influenced by purchase experience and customer satisfaction, with satisfaction serving as a mediating variable. The fundamental dimensions of perceived value typically include functional factors such as product quality, cost-effectiveness, and practicality. When consumers perceive cultural and creative products to meet or exceed expectations in these fundamental attributes (e.g., high-quality materials and reasonable pricing), it directly enhances satisfaction. As satisfaction functions as a psychological feedback mechanism, it further reduces perceived decision risk and strengthens purchase intention. For instance, when purchasing stationery, consumers may develop satisfaction due to “good value for money” when the writing function is stable and the design practical, leading to increased willingness to repurchase or try other products from the same brand. Consequently, the transformation of functional value into behavioral tendency via emotional cognition is consistent with the theoretical framework of “perceived value → emotional attitude → behavioral intention.” The following hypotheses are thus proposed:

*H5: Customer satisfaction mediates the relationship between perceived value and purchase intention;*

*H5a: Customer satisfaction mediates the relationship between functional quality value and purchase intention;*

*H5b: Customer satisfaction mediates the relationship between functional price value and purchase intention;*

*H5c: Customer satisfaction mediates the relationship between emotional value and purchase intention;*

*H5d: Customer satisfaction mediates the relationship between social value and purchase intention.*

Perceptual innovation is reflected in consumers recognition of the uniqueness, novelty, and creative expression in cultural and creative products. In accordance with the diffusion theory of innovation, innovative attributes have the capacity to stimulate curiosity and the desire to experiment; however, these attributes must be mediated through satisfaction in order to translate into actual purchasing intent. For instance, if a cultural product demonstrates breakthroughs in design, technology, or cultural integration (such as combining augmented reality technology with traditional craftsmanship), consumers will develop an initial interest due to “novel experiences.” However, should the product prove satisfactory during its utilization, satisfaction levels are known to increase significantly, thus encouraging repeat purchases and positive word-of-mouth promotion. As Saha [36] observed, the assertion that “experience influences satisfaction” is consistent with this rationale. Innovation must be filtered through satisfaction to circumvent the adverse effects engendered by “curiosity followed by disappointment”.

*H6: Customer satisfaction mediates the relationship between perceptual innovation and purchase intention;*

The concept of cultural perception in the context of consumer behavior refers to the recognition by consumers of the cultural essence, historical connections, or identity symbolism that is embedded in cultural and creative products. As the fundamental characteristic of such products, cultural attributes may primarily influence purchase intentions through emotional resonance and satisfaction mediation. For instance, the procurement of Palace Museum-themed merchandise may be enhanced by designs that accurately convey traditional cultural quintessence, such as symbolic patterns and narrative contexts. These designs have the potential to evoke cultural pride and a sense of belonging, thereby enhancing satisfaction. Conversely, high satisfaction has been shown to reinforce consumers respect for cultural values and to support the encouragement of emotional connections through purchases. This finding is consistent with Dash's [34] theory of “cultural identity driving loyalty,” which posits that cultural attributes must be translated into sustained purchasing intent through satisfaction. It is proposed that the following hypotheses be put forward on the basis of the analysis that has been conducted.

*H7: Customer satisfaction mediates the relationship between perceptual culturality and purchase intention.*

**Fig 2:**
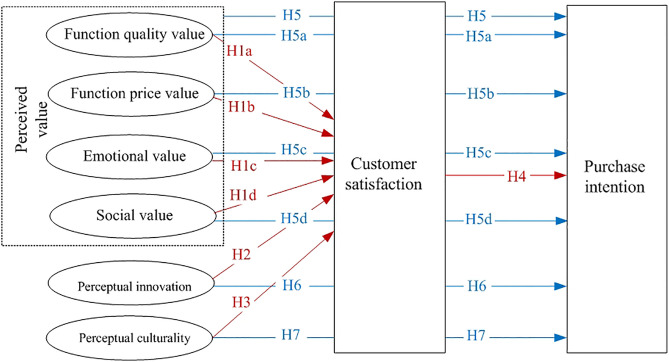
Perception value equation model.

Based on the above analysis, the theoretical model is shown in Fig 2.

## 4. Research design

### 4.1. Questionnaire design and data collection

#### 4.1.1. Questionnaire design.

The questionnaire, which was designed for the purposes of this study, comprises 38 items, the majority of which primarily investigate consumers’ basic personal information and their willingness to purchase cultural and creative products. In relation to the scale design for measuring consumers’ purchasing intention, this paper draws upon extant academic research findings and establishes eight dimensions [2,3,31,36], including functional quality, value, functional price value, and emotional value. The dimensions under scrutiny encompass 26 observation variables, with the utilisation of Likert 7-point scales for the purpose of dimension scoring. The specific measurement items are shown in Table 1.

**Table 1:** Dimension division and measurement item setting of the scale.

Dimension	Reference	Measurement items
Functional quality value	Sweeney et al. [3]	Q1: I will purchase cultural and creative products that are reliable in craftsmanship and durable.
		Q2: I will purchase cultural and creative products of a certain quality.
		Q3: I will purchase cultural and creative products with very good texture (materials, textures).
		Q4: I will purchase cultural and creative products with practical functions.
Function price value	Sheth et al. [2]	Q5:I will purchase cultural and creative products at reasonable prices.
		Q6: I will purchase cost-effective cultural and creative products.
		Q7: I will purchase cultural and creative products that offer good value for money.
Emotional value	Saha S Ket al. [36]	Q8: Cultural and creative products can make me feel novel.
		Q9: Cultural and creative products can evoke emotional resonance in me.
		Q10: Cultural and creative products can bring me entertainment.
Social value	Sweeney et al. [3]	Q11: Cultural and creative products can help me leave a good impression on others.
		Q12: Cultural and creative products can showcase one's personal taste.
		Q13: Cultural and creative products can be given to others to promote emotional communication.
Perceptual innovation	Wikhamn, Wajda et al. [31]	Q14: I think cultural and creative products are innovative.
		Q15: I think cultural and creative products have unique and beautiful designs.
		Q16: I think the content and form of cultural and creative products are very novel and interesting.
		Q17: I think cultural and creative products are brands with personality.
Perceptual culturality	Sheth et al. [2]	Q18: Cultural and creative products can provide me with a way to access historical and cultural information.
		Q19: Cultural and creative products can deepen my understanding of a certain type of culture.
		Q20: Cultural and creative products can ignite my enthusiasm for cultural learning.
Customer satisfaction	[3]	Q21: Purchasing cultural and creative products was a correct decision.
		Q22: I’m very satisfied with the overall design of the cultural and creative products.
		Q23: I think the satisfaction with cultural and creative products has exceeded my expectations.
Purchase intention	Saha S Ket al. [36]	Q24: I am willing to purchase cultural and creative products.
		Q25: Purchasing cultural and creative products is a very good idea.
		Q26: If necessary, I will purchase cultural and creative products again.

#### 4.1.2. Sample characteristic distribution.

The present study conducted a pilot survey from 3^(rd) April to 15^(th) April 2025, focusing on four dimensions: fundamental characteristics of cultural and creative products, unique attributes of these products, customer satisfaction, and purchase intention. Following the identification of issues during the pilot phase, the questionnaire was revised and refined. The formal survey was conducted from April 20 to May 10, 2025. It is important to note that, due to the feasibility of the study and the availability of resources, this research employed convenience sampling, selecting multiple museums in Xi'an and Baoji cities, Shaanxi Province, as study sites. A total of 333 valid questionnaires were collected, achieving a 93.8% response rate. The descriptive statistics of the sample demographics are presented in Table 2.

**Table 2:** Descriptive statistics of samples.

Variable	Options	Frequency	Proportion(%)
Gender	Male	181	54.4
	female	152	45.6
	Under 18 years old	42	12.6
	Aged 18–25	101	30.3
Age	Aged 26–35	136	40.8
	Aged 36–45	29	8.7
	Aged 46–55	23	6.9
	Over 56 years old	2	0.6
Occupation	Student	117	35.1
	Personnel of public institutions	33	9.9
	Government agencies	25	7.5
	Individual business operation	21	6.3
	Enterprise staff	60	18
	Freelancer	32	9.6
	Others	45	13.5
Monthly disposable income	Less than 1,000	78	23.4
	From 1000 to 2999	84	25.2
	3,000–4,999	93	27.9
	5,000–6,999	34	10.2
	More than 7,000	44	13.2
The price range for purchasing cultural and creative products	Under 60	131	39.3
	61–100	158	47.4
	101–300	141	42.3
	301–600	30	9
	601–1000	6	1.8
	More than 1,000	7	2.1

### 4.2. Credibility and validity analysis

In order to ensure the reliability and validity of the proposed scale, this study conducted a reliability and validity analysis using SPSS 25.0 software, with specific results shown in Table 3. The Cronbach’s α coefficient is a measure of internal consistency across dimensions. A higher Cronbach’s α value is indicative of enhanced internal consistency, with coefficients exceeding 0.7 typically deemed reliable. As demonstrated in Table 3, the Cronbach’s α values for each dimension range from 0.804 to 0.925, while the overall Cronbach’s α coefficient reaches 0.918, thus demonstrating excellent internal consistency. Furthermore, the KMO value is 0.884, and the Blattlett test demonstrating significant p-values (Sig. < 0.05), thereby confirming the suitability of the sample data for factor analysis.

**Table 3:** Results of scale reliability and validity analysis.

Variable	Measurement items	Load STD	Cronbach’s α coefficient	CR	AVE
Functional quality value	Q1	0.716	0.829	0.831	0.552
	Q2	0.792			
	Q3	0.721			
	Q4	0.740			
Function price value	Q5	0.672	0.804	0.810	0.590
	Q6	0.880			
	Q7	0.737			
Emotional value	Q8	0.804	0.837	0.839	0.634
	Q9	0.832			
	Q10	0.751			
Social value	Q11	0.758	0.822	0.820	0.604
	Q12	0.837			
	Q13	0.732			
Perceptual innovation	Q14	0.830	0.872	0.872	0.630
	Q15	0.801			
	Q16	0.768			
	Q17	0.775			
Perceptual culturality	Q18	0.755	0.826	0.829	0.618
	Q19	0.808			
	Q20	0.795			
Customer satisfaction	Q21	0.889	0.917	0.917	0.786
	Q22	0.880			
	Q23	0.891			
Purchase intention	Q24	0.922	0.925	0.926	0.806
	Q25	0.854			
	Q26	0.916			

The present study employs Structural Equation Modeling (SEM) for model validation, a technique that has the capacity to address latent variables, measurement errors and multiple relationships simultaneously. Initially, confirmatory factor analysis was conducted using Amos software to validate the model's validity. As demonstrated in Table 3, factor loadings ranged from 0.672 to 0.922, all exceeding the standard threshold of 0.5. This finding suggests that latent variables possess considerable explanatory power over measured variables, accompanied by reliable indicator reliability. The composite reliability (CR) values for each dimension ranged from 0.810 to 0.926, consistently above 0.7. The variance explained (AVE) values also fell within the 0.552–0.806 range, with all values exceeding 0.5. The findings, when considered collectively, demonstrate robust convergent validity and composite reliability across all dimensions.

The results of the discriminant validity test for each dimension of the scale are shown in Table 4. The square root values of the standardized correlation coefficients between each pair of dimensions are lower than those corresponding to the square root values of the AVE of each dimension. This indicates that the dimensions of the scale have good discriminant validity.

**Table 4:** Validity test results of dimension differences in the scale.

	(1)	(2)	(3)	(4)	(5)	(6)	(7)	(8)
Purchase intention	0.806							
Satisfaction	0.381	0.786						
Perceptual culturality	0.342	0.558	0.618					
Perceptual innovation	0.319	0.547	0.484	0.63				
Social value	0.32	0.517	0.406	0.45	0.604			
Emotional value	0.286	0.596	0.515	0.459	0.504	0.634		
Function price value	0.27	0.504	0.303	0.304	0.358	0.473	0.59	
Functional quality value	0.252	0.493	0.333	0.265	0.491	0.394	0.43	0.552
Square root of AVE	0.898	0.887	0.786	0.794	0.777	0.796	0.768	0.743

## 5. Empirical analysis

### 5.1. Model fitness test

The external validity of the constructed structural equation model (SEM) was evaluated by employing multiple indicators, including GFI, TLI, and RMSEA, to assess model fit. The specific test results are displayed in Table 5. The chi-square freedom ratio (X^2^/df) was 1.552, falling within the 1–3 range. The fit indices (GFI, CFI, and TLI) exceeded 0.9 (with values of 0.909, 0.969, and 0.963, respectively). The root mean square error of approximation (RMSEA) and the standardised root mean square residual (SRMR) were 0.041 and 0.044, respectively, suggesting that the overall model exhibited excellent fit.

**Table 5:** Results of structural equation model fit test.

Appropriate indicators	Measured value	Reference value criteria	Result
X²/df	1.552	0 < X²/df < 5	perfect
GFI	0.909	>0.9	perfect
AGFI	0.885	<0.9	tolerableness
CFI	0.969	>0.9	perfect
TLI	0.963	>0.9	perfect
RMSEA	0.041	<0.1	perfect
SRMR	0.044	<0.08	perfect

### 5.2. Model path relationship test

**Fig 3:**
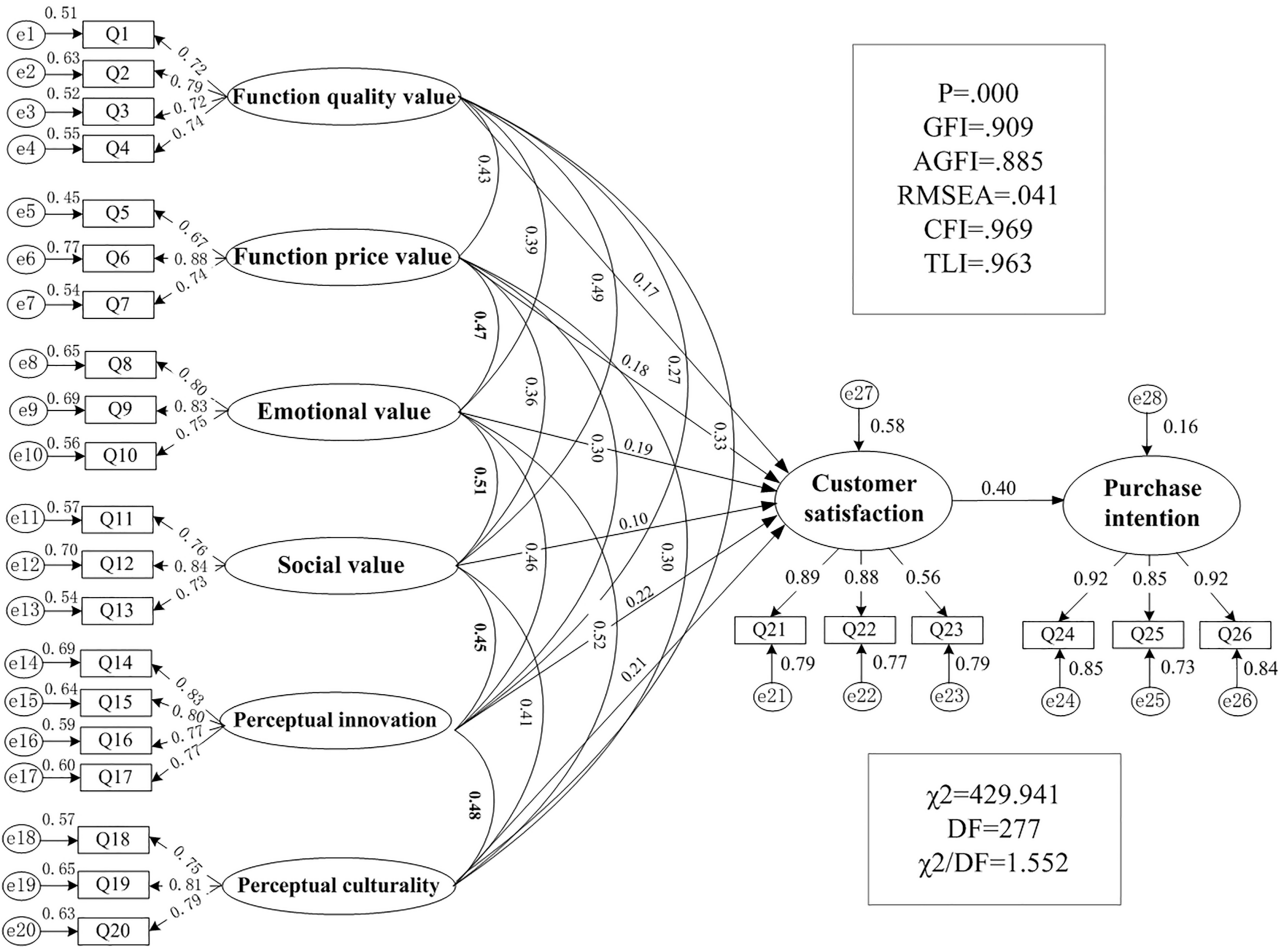
Path coefficient diagram of customer purchase intention based on perceived value theory.

The construction of a structural equation model of customers purchase intention was based on the theoretical framework of perceived value theory, as illustrated in Fig 3.

The present study employed structural equation modelling to validate the pathways through which perceived value dimensions influence the purchase intention of cultural and creative products (see Fig 3). Initially, hypothesis testing was conducted (H1-H4) utilising the Amos software. As illustrated in Table 6, the reader will find path coefficients, significance levels, and hypothesis test results. The findings indicate that, in addition to social value, functional quality, functional price, emotional value, perceptual innovation, and perceived cultural value all significantly positively affect customer satisfaction and purchase intention. It is noteworthy that perceptual innovation (β = 0.221) demonstrates the most significant impact, even surpassing emotional value (β = 0.189). This finding is consistent with the mounting emphasis on “experience-driven” and “cognitive engagement” in contemporary consumer behaviour. Specifically, the standardized path coefficients for hypotheses H1a, H1b, H1c, H2, and H3 are all greater than 0 with P-values below 0.05, thus confirming statistically significant positive correlations. These results provide validation for hypotheses H1a through H3. However, the P-value for H1d (0.139) remains significantly higher than the 0.05 threshold, indicating that social value does not significantly predict customer satisfaction. The study further corroborates the notion that consumer satisfaction with cultural and creative products can significantly influence purchase intention, thereby validating hypothesis H4.

**Table 6:** Results of hypothesis testing for SEM path relationships.

Hypothesis	Path relationship	S.E.	C.R.	P	Standardized regression coefficient
H1a	Functional quality value→Satisfaction	0.079	2.835	0.005	0.166
H1b	Function price value→Satisfaction	0.089	3.19	0.001	0.181
H1c	Emotional value→Satisfaction	0.086	2.838	0.005	0.189
H1d	Social value→Satisfaction	0.081	1.48	0.139	0.099
H2	Perceptual innovation→Satisfaction	0.073	3.812	*	0.221
H3	Perceptual culturality→Satisfaction	0.066	3.448	*	0.209
H4	Satisfaction→Purchase intention	0.056	7.047	*	0.398

On the one hand, the presence of varied experiences has the potential to counteract the issue of homogenisation. The integration of novel designs with multidimensional experiences, encompassing visual and interactive elements, has been demonstrated to elicit a heightened level of consumer curiosity. This, in turn, has been shown to activate brain reward mechanisms and facilitate the formation of enduring memory anchors. For instance, the Dunhuang Academy's “Digital Donor” project employs augmented reality (AR) technology to engage users in mural restoration, transforming static cultural symbols into interactive narrative scenarios that prolong user engagement. Conversely, the process of cultural encoding and decoding has been demonstrated to engender a state of mutual empowerment. Contemporary innovative cultural translation has been shown to convert obscure historical symbols into consumable symbolic capital, thereby allowing consumers to achieve intellectual fulfilment during decoding processes and form psychological compensation through “cultural knowledge growth”. This positive cycle of innovation and satisfaction establishes a new consumption paradigm driven by “cultural-technological dual-wheel propulsion.” Products have evolved beyond their practical functions, becoming cognitive carriers that embody cultural genes, stimulate creative potential, and connect intergenerational memories. This is precisely the hallmark of cultural consumption upgrades in modern society.

Furthermore, social value did not demonstrate significant influence in this study. This finding may be indicative of a shift in the cultural and creative consumption sphere, particularly among younger, more educated demographics, where the importance of personalised experiences, self-expression, and intrinsic cultural identity has superseded traditional motivations for social prominence and group affiliation. In contemporary society, consumers are increasingly prioritising cultural consumption as a means of enriching their personal world of meaning. This shift in focus is marked by a shift in perception, whereby cultural consumption is no longer primarily viewed as a tool for conveying social class symbols.

In order to verify further whether customer satisfaction plays a mediating effect on the influence of perceived value on purchase intention, the Bootstrap mediation effect test was used for verification. The results of the study are presented in Table 7.

**Table 7:** Mediation tests based on customer satisfaction.

Path relationship	Effect	SE	Bias Corrected (95%)			Percentile method (95%)		
			LLCI	ULCI	P	LLCI	ULCI	P
Functional quality value – Satisfaction – Purchase intention	0.066	0.032	0.010	0.135	0.019	0.007	0.133	0.022
Function price value – Satisfaction – Purchase intention	0.072	0.028	0.024	0.136	0.002	0.023	0.133	0.003
Emotional value – Satisfaction – Purchase intention	0.075	0.034	0.013	0.148	0.014	0.013	0.149	0.014
Social value – Satisfaction – Purchase intention	0.040	0.047	−0.048	0.135	0.357	−0.054	0.130	0.429
Perceptual innovation – Satisfaction – Purchase intention	0.088	0.029	0.038	0.151	0.001	0.035	0.147	0.002
Perceptual culturality -Satisfaction – Purchase intention	0.083	0.032	0.027	0.155	0.002	0.028	0.156	0.002

As demonstrated in Table 7, the mediation effect of customer satisfaction on perceived value in Hypothesis H5 is statistically significant. The mediating effects for functional quality, functional price, and emotional value are 0.066, 0.072, and 0.075 respectively, with P-values below 0.05 at the 95% confidence interval. However, the mediating effect for social value is 0.040, with P-values above 0.05 at the same confidence level. The findings of this study corroborate Hypotheses H5a, H5b, and H5c, with the exception of the mediating effect on social value, as postulated by Hypothesis H5d. The results of Hypothesis H6 demonstrate a significant mediating effect (P < 0.005) between perceptual innovation and purchase intention, thus supporting Hypothesis H6. The results of Hypothesis H7 indicate a significant mediating effect (P < 0.002) between perceived cultural value and purchase intention, thereby confirming the hypothesis. The results of hypothesis testing are presented in Table 8.

**Table 8:** Hypothesis test result.

Hypothesis	Path relationship	Coefficients	Whether to accept the null hypothesis
H1a	Functional quality value→Satisfaction	0.166	Yes
H1b	Function Price value→Satisfaction	0.181	Yes
H1c	Emotional value→Satisfaction	0.189	Yes
H1d	Social value→Satisfaction	0.099	No
H2	Perceptual innovation→Satisfaction	0.221	Yes
H3	Perceptual culturality→Satisfaction	0.209	Yes
H4	Satisfaction→Purchase intention	0.398	Yes
H5a	Functional quality value – Satisfaction – Purchase intention	0.066	Yes
H5b	Function price value – Satisfaction – Purchase intention	0.072	Yes
H5c	Emotional value – Satisfaction – Purchase intention	0.075	Yes
H5d	Social value – Satisfaction – Purchase intention	0.04	No
H6	Perceptual innovation – Satisfaction – Purchase intention	0.088	Yes
H7	Perceptual culturality -Satisfaction – Purchase intention	0.083	Yes

## 6. Research conclusions and deficiencies

In order to address the prevalent issues of homogenization, repetitive symbolism, and insufficient substantive innovation in cultural and creative products, this study establishes an integrative theoretical framework based on product value attributes, proposing a cascading mechanism: “perceived value → customer satisfaction → purchase intention.” The validity of this framework is validated through empirical testing of research hypotheses using structural equation modeling, which systematically reveals the underlying mechanism by which perceived value influences purchase intention through satisfaction. The study conducts an in-depth analysis of key factors affecting consumer purchase intention from both value attributes and product characteristics, thereby providing targeted recommendations for improving the development and marketing practices of museum cultural and creative products. The primary research findings are as follows:

(1) The H1a and H1b results demonstrate that the quality and price of cultural and creative products significantly impact customer satisfaction, indicating that consumers prioritize cost-effectiveness and functional practicality when purchasing such items. Contemporary young consumers have evolved their expectations regarding products from merely pursuing cost savings to meeting fundamental quality standards. These include tactile comfort, material safety, and esthetic craftsmanship, reflecting diversified value preferences. Consequently, while preserving artistic and visual appeal in cultural and creative product design, priority should be given to ensuring functionality and quality.
(2) The H1c hypothesis demonstrates that the emotional value of cultural and creative products can significantly enhance customer satisfaction. When considering this dimension, designers should incorporate their unique characteristics and symbolic elements into product design, enabling consumers to develop emotional resonance with the products during usage scenarios. However, the H1d hypothesis reveals no significant impact from social value: customers primarily purchase these products for personal needs rather than pursuing social significance. This finding indicates that design evaluations may be able to play down social value metrics in the context of product development.
(3) The results of Hypothesis H2 and H3 demonstrate that perceptual innovation and cultural relevance significantly enhance customer satisfaction. This finding serves to corroborate Drucker‘s assertion that “the essence of business is creating customers.” In the experience economy era, it is imperative for companies to establish emotional connections through cultural resonance and to create cognitive differentiation via innovative perceptions in order to achieve exponential growth in customer value perception. In order to enhance customer satisfaction, businesses may consider the following strategies: Firstly, the integration of cultural elements through regular launches of differentiated products/services with unique features/designs is recommended in order to strengthen product/service innovation. In addition, the exploration of cultural IP value by transforming regional characteristics and historical elements into tangible consumer experiences is advised. Secondly, the innovation journey and cultural DNA behind products must be presented visually through brand storytelling videos and immersive exhibitions. Furthermore, the development of cultural knowledge and the provision of value-added services (for example, traditional craft instruction and the interpretation of cultural merchandise) should be considered to enhance cognitive fulfillment during the consumption process.
(4) The results confirm Hypothesis 4. It indicates that an increase in customer satisfaction leads to a weakening of consumers’ perceived risk of decision-making, which can stimulate their purchase intention. This is achieved through emotional identification with cultural and creative products, and the establishment of brand trust.
(5) The findings of the benchmark regression analysis indicate that perceptual innovation and cultural cognition exert the most substantial influence on customer satisfaction. Subsequent mechanism analysis demonstrates that customer satisfaction serves as a significant mediating factor between these two dimensions. Therefore, from the perspective of perceived innovativeness, culture, emotion and functionality are the primary distinguishing factors among different cultural and creative products. Designers must extract traditional cultural elements, artistic symbols and color schemes based on user needs, whilst updating product design approaches to achieve artistic innovation. Incorporating traditional cultural elements necessitates the minimization of subjective intervention and the prioritization of alignment between fundamental functional requirements and user usage scenarios. In relation to cultural perception, it is incumbent upon designers to undertake a thorough exploration of the fundamental principles and the inherent value of traditional culture, with a view to enhancing the recognizability of the product. During the design process, it is possible to undertake a vertical analysis of regional characteristics, architecture, patterns, and artifacts, while simultaneously establishing horizontal connections between related classic stories and cultural heritage elements. This approach is intended to prevent issues such as homogenization and symbolic repetition.

The innovations of this study are reflected in the following aspects: Firstly, this study transcends the limitations of traditional perceived value models through contextual expansion and dimensional integration. The integration of the two core attributes of cultural and creative products – innovation perception and cultural perception – into the classic CPV framework establishes an integrated analytical model tailored for cultural and creative consumption. This work elucidates the value-driven structure that distinguishes cultural and creative products from ordinary commodities, thereby providing a more essential theoretical tool for related research.

Secondly, this study provides a comprehensive validation of chain-mediated pathways, thereby deepening our understanding of the so-called “value transformation black box”. In contrast to the majority of research that focuses exclusively on direct effects, our study rigorously examined and confirmed the complete mediating role of customer satisfaction in the relationship between “perceived value” and “purchase intention”. The subsequent analysis sought to elucidate the manner in which disparate perceived dimensions- namely, functional quality value, functional price value, emotional value, perceptual innovation, and perceived cultural value – exert an indirect influence on purchase intention through the medium of satisfaction. This provides a clear mechanistic explanation for how cultural value translates into actual consumer behaviour. The conclusions provide insights for the marketing of cultural and creative products: designers should balance functionality, innovativeness, and cultural value, and achieve the transformation from “interest” to “action” by enhancing satisfaction.

Thirdly, this study provides a contextualised revision of extant theoretical assumptions through an empirical reflection on the role of “social value.” The findings of the study indicate that in the context of museum cultural and creative consumption, social value exerts minimal influence on satisfaction or purchase intention. This finding suggests that consumers’ purchasing motivation may stem more from intrinsic cultural identity and personal experiences than from external social display needs, prompting a re-examination of the boundaries and applicability of social value in cultural consumption.

Nevertheless, the present study is not without its limitations. In terms of research depth, the research model primarily focuses on intrinsic product attributes while neglecting consumer characteristics and purchasing environments, which introduces inherent constraints. It is recommended that future research efforts focus on the development of a comprehensive “innovation-cultural-satisfaction” framework. This framework should incorporate moderating variables, such as individual consumer traits (e.g., age and cultural background) and product categories. The integration of these moderating variables will facilitate the identification of differential impact pathways, which will contribute to a more nuanced understanding of the factors influencing consumer behavior. In addition, the incorporation of contextual factors, such as customer purchase scenarios for cultural and creative products, in conjunction with additional evaluation metrics, would serve to provide more scientifically grounded theoretical foundations for their design and development.

In terms of research design, the present study focuses on the audience groups of multiple museums in Shaanxi Province as the research object. While this approach effectively unveils the cognitive processes of consumers within the confines of this particular cultural context, it concomitantly imposes limitations on the extent to which the conclusions can be generalised. The rationale behind this phenomenon is attributable to the considerable size of China, accompanied by pronounced disparities in economic development, cultural heritage, and audience composition across various regions. It is imperative that future research is validated and compared in a broader geographical area and across a wider range of museum types. Moreover, the present study was chiefly concerned with conducting a cross-sectional study, the result of which was that consumer cognition was captured solely at the time of data collection. In reality, the public’s perception of museum cultural and creative products is a dynamic process, easily influenced by cultural events, market trends, and policies. Consequently, future research should be directed towards two primary areas. Firstly, the execution of cross-regional comparative studies to investigate the impact of differing socio-economic and cultural factors on consumer cognition. Secondly, the adoption of a longitudinal research design to monitor the progression of consumer cognition over time, thereby yielding more dynamic and in-depth insights.

## Supporting information

S1 Data Raw data. (XLSX)
